# In Silico Drug Screening Based Development of Novel Formulations for Onychomycosis Management

**DOI:** 10.3390/gels7040221

**Published:** 2021-11-18

**Authors:** Mahak Fatima, Sadia Monawwar, Sradhanjali Mohapatra, Thomson Santosh Alex, Abdulrahman Ahmed, Mohamad Taleuzzaman, Asgar Ali, Mohammad Javed Ansari, Mohd. Aamir Mirza, Zeenat Iqbal

**Affiliations:** 1 Department of Pharmaceutics, School of Pharmaceutical Education and Research, Jamia Hamdard, New Delhi 110062, India; Mahak5010@gmail.com (M.F.); sadiamonawwar2@gmail.com (S.M.); sibanee@gmail.com (S.M.); thomsonsantoshalex@gmail.com (T.S.A.); abd.hamdani94@gmail.com (A.A.); aali@jamiahamdard.ac.in (A.A.); 2 Department of Pharmaceutical Chemistry, Faculty of Pharmacy, Maulana Azad University, Village Bujhawar, Tehsil Luni, Jodhpur 342802, India; zzaman007@gmail.com; 3 Department of Pharmaceutics, College of Pharmacy, Prince Sattam Bin Abdulaziz University, Al-kharj 16278, Saudi Arabia; mj.ansari@psau.edu.sa

**Keywords:** amorolfine HCl, vitamin E, undecylenic acid, nail lacquer, nanoemulgel, Box–Behnken design, transungual permeation

## Abstract

Onychomycosis is a prominent fungal infection that causes discoloration, thickening, and mutilation leading to the separation of the nail from the nail bed. Treatment modalities for onychomycosis may include oral, topical, or combination therapy with antifungals and at times may require chemical or surgical intervention. The burden of side effects of antifungals is enormous, and therefore using molecular docking-based drug selection in context with the target keratin protein would ensure better disease management. Ciclopirox, Amorolfine HCl, Efinaconazole, Tioconazole, and Tavaborole were submitted for assessment, revealing that Amorolfine HCl is the best fit. Consequently, two formulations (Nail lacquer and nanoemulgel) were developed from Amorolfine HCl to validate the in silico screening outcomes. The formulations were further fortified with over-the-counter ingredients vis-a-vis with vitamin E in nail lacquer and undecylenic acid in nanoemulgel for their prominent roles in improving nail health. Both the formulations were systematically designed, optimized, and characterized. Amorolfine HCl containing nanoemulgel (NEG) was developed using undecylenic acid as an oil phase and thioglycolic acid as a penetration enhancer. The quality parameters evaluated were particle size, the zeta potential for nanoemulsion (NE) (78.04 ± 4.724 nm and −0.7mV, respectively), in vitro cumulative drug release (96.74% for NE and 88.54% for NEG), and transungual permeation (about 73.49% for NEG and 54.81% for NE). Nail lacquer was evaluated for the drying time, non-volatile content, and blush test. In vitro cumulative drug release of the developed nail lacquer and comparator marketed formulations were around 81.5% and 75%, respectively. Similarly, the transungual drug permeation was 6.32 μg/cm^2^ and 5.89 μg/cm^2^, respectively, in 24 h. The in silico guided preparation of both formulations containing Amorolfine HCl and over the counter ingredients is amenable for therapeutic use against onychomycosis and will be evaluated in the in vivo model.

## 1. Introduction

Onychomycosis, a fungal infection that occurs in nails and may spread to the neighbouring skin cells, is characterized by discoloration and thickening of nail plates and onycholysis [1]. It is one of the most prominent nail infections found throughout the world, accompanying many other complications such as local pain, discomfort, and disfigurement, producing severe physical and occupational limitations, thus leading to a decline in the quality of life for the affected population. Generally, onychomycosis is caused by the pathogenic fungi *Trichophyton rubrum*, *Trichophyton mentagrophytes*, and *Epidermophyton*. Often, this infection begins from Tinea pedis, a fungal infection commonly observed in the surrounding skin of the feet. Nail trauma, occlusive footwear, humidity, sweat, and sometimes genetic predisposition led to the progression of the disease. A robust immune system can fight this infection, but patients with diabetes, poor peripheral circulation, HIV, and immunosuppression have higher chances of infection [2,3].

Onychomycosis can cause severe complications in diabetic patients and should be treated immediately when early symptoms appear. Approximately one-third of the global diabetics population is affected by this disease and factors such as age, male gender, obesity, poor glycaemic control, and peripheral vascular diseases are associated with higher rates of infection. If not treated properly, it can prove to be a limb threatening infection. Along with this, co-existence of other medical conditions and drug–drug interactions are the drawbacks of various oral anti-fungals, which makes the treatment regimen selection more complicated in diabetics [4].

The treatment of this disease is complex because of the impermeable nature of the nail and entrenched nature of the infection, along with a long-duration of treatment, poor patient adherence, and frequent recurrences. Current treatments cannot show complete efficacy for many reasons, such as slow nail growth, limited penetration of topical drugs, adverse effects of oral drugs, etc. Previous research in this area has reported several innovative therapeutic approaches for its treatment [5]. There is a multitude of factors such as the extent and severity of nail changes, infecting organism, drug interaction, adverse effects problems, and history of success or failure of previous treatments that determine the type of treatment to be initiated [6].

The infection assessment is based on the degree of nail involvement, area of nail discoloration and nail plate thickening, separation of the nail from the nail bed (onycholysis), and pain. A minor infection is treated through topical application of medicines like Amorolfine and Ciclopirox. The advantage of topical treatments is fewer side effects. However, the disadvantage is that the duration of treatment is long, and the efficacy is limited due to poor nail plate penetration [7]. The nail’s structure is highly stable due to strong disulfide linkages and hydrogen bonds in the keratin network, making it the most formidable biological barrier [8]. Thus, onychomycosis treatment choices are minimal because of deep-seated infection and the impermeable nature of nails.

Researchers have recently focused on enhancing topical delivery for the effective treatment of onychomycosis. The marketed formulation of Amorolfine and Ciclopirox nail lacquers are generally used for the treatment.

Amorolfine has been reported as an effective treatment for onychomycosis. Belonging to the morpholine group of synthetic antifungals, Amorolfine exhibits broad-spectrum fungistatic as well as fungicidal activity. Its mechanism of action includes inhibition of delta-14-reductase and delta-8 and delta-7 isomerase enzymes in the ergosterol biosynthesis pathway. It exhibits fungicidal activity against *T. mentagrophytes* and *C. albicans*. It is available as a 5% nail lacquer and is applied once or twice a week for 6– 12 months and it further persists in the nail for 14 days after the completion of the treatment. Clinical studies have shown that once a week application has a mycological cure rate of 70.6% and a complete cure rate of 46%. At the same time, a twice a week application has a mycological cure rate of 76.1% and a complete cure rate of 54.2%. Additionally, side effects with amorolfine nail lacquer are rare and limited to local burning, pruritus, and erythema [9].

A list of widely used products available in the US market is given in Table 1, including over the counter (OTC) products. With the advancement of techniques and the introduction of novel drug delivery systems such as nanoparticles, microemulsions, polymeric films, and nail lacquers, it became easy to impart quality characteristics such as superior drug permeation and localized action [10]. The penetrability of drugs also increases by the development of gel formulation [11,12]. Furthermore, the Quality by Design approach for optimizing formulation methods has several advantages such as reducing the number of experiments, cost-effectiveness, and considerably less laboratory work [13]. Furthermore, it has a scope to obtain the design space for scale-up and is preferably used for designing and developing various formulations. Molecular docking has been exploited for selecting suitable drugs which could target the selected protein moiety and hitherto accentuate the therapeutic outcomes. Based on this, the currently marketed drugs were investigated using in silico screening. The best fit molecule was then zeroed and employed to prepare different novel formulations (containing OTC components also). This way, we could expect a range of novel formulations with better quality characteristics and therapeutic outcomes.

Further, NEG (nanoemulgel) is a novel strategy that can provide desired therapeutic effect efficiently in less time, with good patient compliance. Despite several advantages such as thixotropy, greaselessness, easy spreadability, emolliency, and non-staining nature [1]. NEG is also a suitable carrier for lipophilic drugs to the deeper layers of the nail. The bioavailability of lipophilic drugs can be increased by incorporating them into the oil phase of the emulsion and then converting them into a gel formulation with better stability and prolonged release kinetic. We followed the same procedure for formulating a topical preparation incorporating Amorolfine HCl that can be easily applied and will readily absorb via transungual route, thus avoiding associated adverse effects that occur in other routes. Additionally, the incorporation of nanotechnology will ensure efficient site-specific drug release over a longer duration. Additionally, undecylenic acid used as an OTC compound in NEG is itself an antifungal agent and has proven to treat superficial infections [14]. On the other hand, the dual component loaded nail lacquer comprising of hydrophilic polymer compactly protected by occlusive hydrophobic polymer is proposed. Hydrophobic polymer due to the lower content of charged groups shows less drug permeability than hydrophilic polymer. Contrary to this, hydrophilic polymers cause more hydration (swelling) of the nail plate as they contain large amounts of water which helps to penetrate the drug. The proposed nail lacquer will assure good penetrability of the Amorolfine HCl with prolonged stay time by incorporating both hydrophilic and hydrophobic polymers, respectively. Moreover, the presence of vitamin E will help to maintain nail health, thus making it more acceptable [15].

The hypothesis worked well when the two developed formulations, nanoemulsion gel (containing Undecylenic acid) and Nail lacquer (containing vitamin E), were evaluated qualitatively with promising therapeutic potential.

## 2. Results and Discussion

### 2.1. Molecular Docking Study

Molecular docking was performed to establish the binding ability of the compounds with keratin. The docking scores of all compounds are presented in Table 2. All the compounds were found to exhibit good interaction with the target protein.

**Figure 1:**
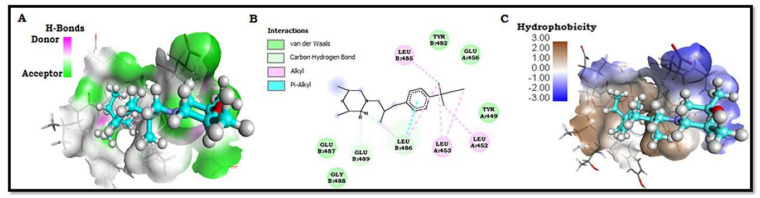
Amorolfine HCl docked structure showing hydrogen bond donor–acceptor residues and hydrophobic surface.

Among all the marketed compounds tested for keratin binding, Amorolfine HCl was found to be most potent and had the highest G score (−7.325 Kcal/mol) compared to other compounds (Table 2). Compound Amorolfine HCl demonstrated two hydrogens bonds-one each with Leu B: 486 and Glu B: 489 residues (Figure 1B). Analysis of the docked structure showed that keratin offers numerous van der Waal, covalent, and electrostatic interactions to Amorolfine HCl compound compared to other compounds, as shown in Table 2 and Figure 1B. Docking procedure suggested that the compound Amorolfine HCl bound with the active site cavity of keratin eliciting hydrogen bond donor–acceptor residues and hydrophobic surface, respectively (Figure 1A,C).

Molecular docking studies suggested that the van der Waals, covalent, carbon–hydrogen, Pi-alkyl, and electrostatic interactions are the key force for holding the compound Amorolfine HCl together with the keratin. Furthermore, compound Amorolfine HCl interactions with keratin supported that the binding of the keratin causes the antifungal and antimicrobial effects of the compound. Compound Amorolfine HCl binds to keratin with an excellent G score, and it may be considered an appreciable inhibitor of fungal and microbial diseases.

### 2.2. Analytical Method Development

A validated analytical method for Amorolfine HCl was developed using UV spectroscopy with a regression coefficient of 0.9999 at 213 nm in methanol having concentration range (2–10 μg/mL) and regression coefficient of 0.998 at 220 nm in PBS pH 7.4 with concentration range (70–100 μg/mL).

### 2.3. Development of Nail Lacquer

#### 2.3.1. Selection of Solvent System Ratio and Optimization Polymer Concentration by Application of Box–Behnken Designs (BBD) to Check the Drying Time and Non-Volatile Content

Nail lacquer was formulated using 32 factorial designs. A total of 17 formulations were prepared, as shown in Table 3, and were evaluated for non-volatile content and drying time.

**Figure 2:**
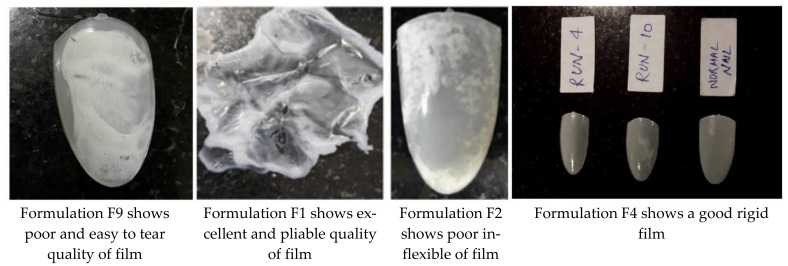
Various qualities of films obtained during optimization of nail lacquer with different ratios of solvents (Ethanol: Water) and polymers (HPC: Eudragit RS).

By the application of BBD, the concentration of both polymers and permeation enhancers were optimized. The best ratio of solvent system (Ethanol: Water) finalized along with polymer ratio (HPC: EUDRAGIT RS100). The ratios of solvent system and polymer ratio were 9:1 and 1:9, respectively. The optimized formulation for drug-free dual-component loaded nail lacquer contains HPC (5 % *w*/*v*), Eudragit 100 RS (5% *w*/*v*), and Ethanol: Water (9:1) mL as depicted in Table 4 and Figure 2.

#### 2.3.2. Preparation of Nail Lacquer

Optimized nail lacquer was prepared using Eudragit RS 100 as hydrophobic polymer, hydroxy propyl cellulose as hydrophilic polymers, triacetin as plasticizer, salicylic acid as a permeation enhancer, and vitamin E as an OTC component.

Vitamin E was incorporated as an OTC compound in the nail lacquer formulation as it has high antioxidant activity in its pure state, which can accelerate the cellular proliferation of fibroblast and epithelial cells, favoring tissue growth; thus, it is effective in mild to moderate distal subungual onychomycosis. In addition, the topical formulation of vitamin E is well absorbed at the level of the nail plate, enhancing tropism of the nail and the periungual tissues, making the nail even more resistant to infections, thus reducing the risk of disease relapse [15].

### 2.4. Evaluation of Nail Lacquer

#### 2.4.1. Drying Time

**Figure 3:**
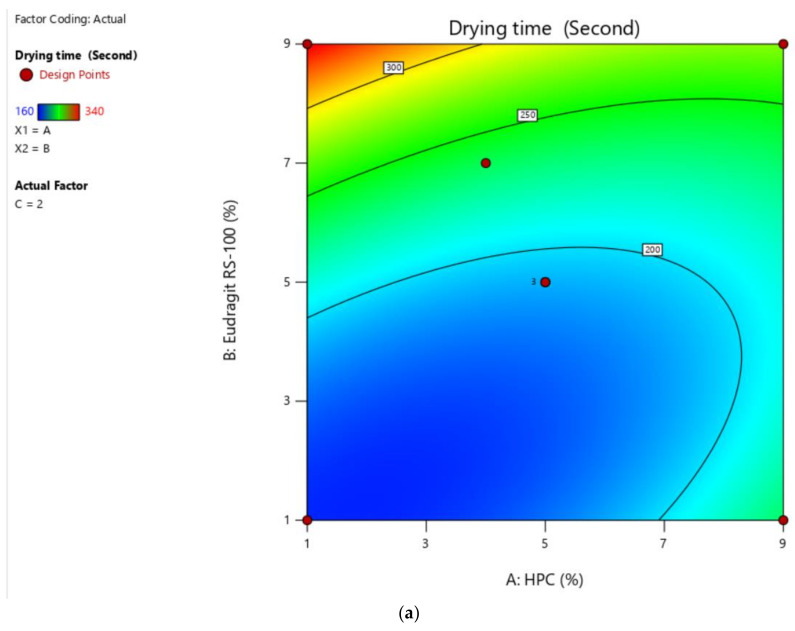

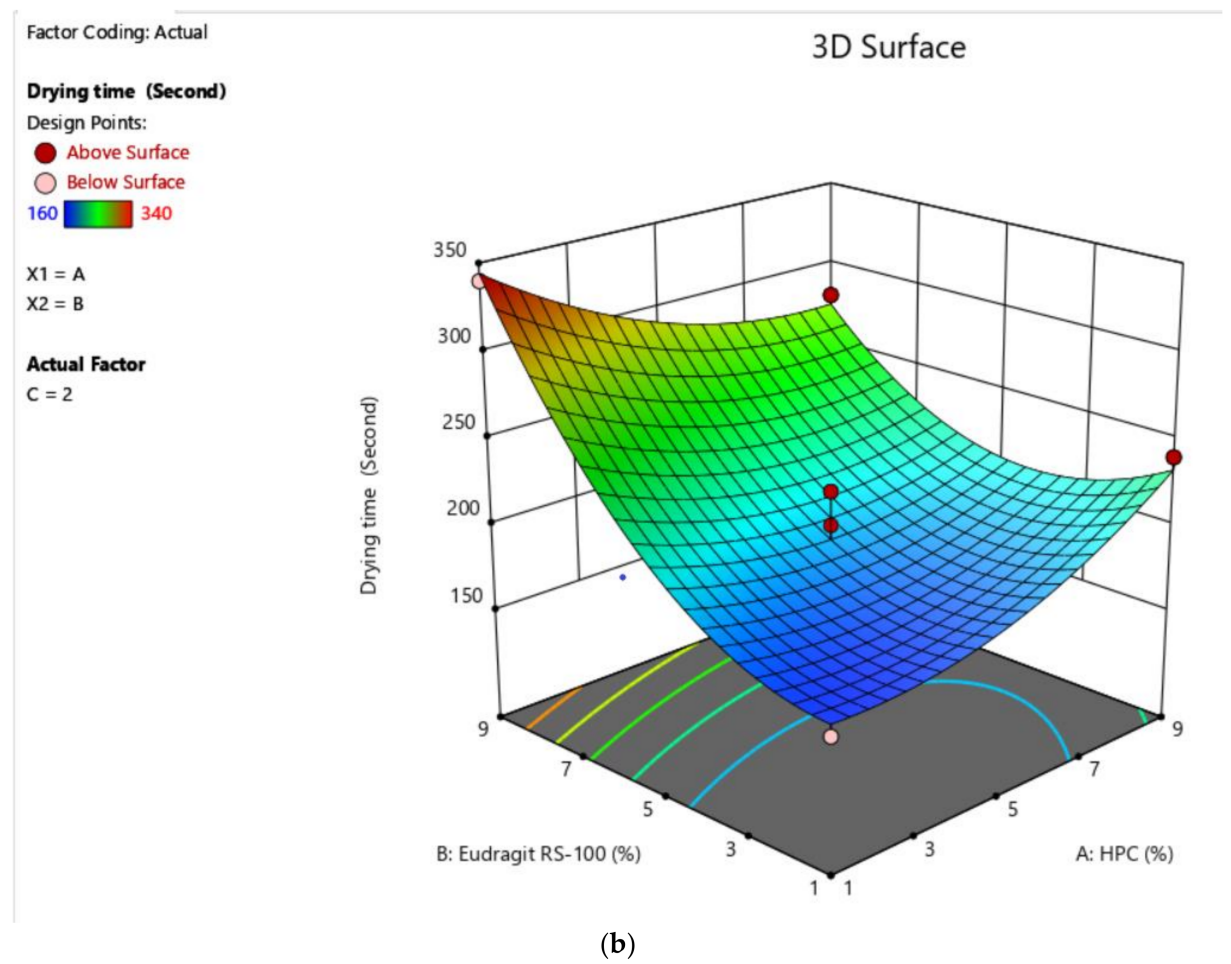
Effect of independent factors on the drying time with contour plot (**a**) and 3-D response surface (**b**) of nail lacquer by the application BBD.

The minimum drying time of the formulation was observed to be 180 s, which was considered good and this formulation was selected as optimal. The effect of variables was studied on drying time. ANOVA suggests that the quadratic model and F-value of 8.48 (*p* < 0.05) implies the model is significant as designated in Table 3 and Figure 3.

#### 2.4.2. Non-Volatile Content

The amount of coverage provided by the film depends on the non-volatile content of the formulation. After solvent evaporation, a solid component is left behind, providing adequate coverage to the entire nail plate. A minimum of 20% by mass of the non-volatile content is required as per the Bureau of Indian Standard, IS 9245: 1994). The non-volatile content for the formulations was observed to be in between 20 to 37%, following the BIS guidelines. The minimum non-volatile content of the formulation was 21%, which was selected for the desired preparation.

**Figure 4:**
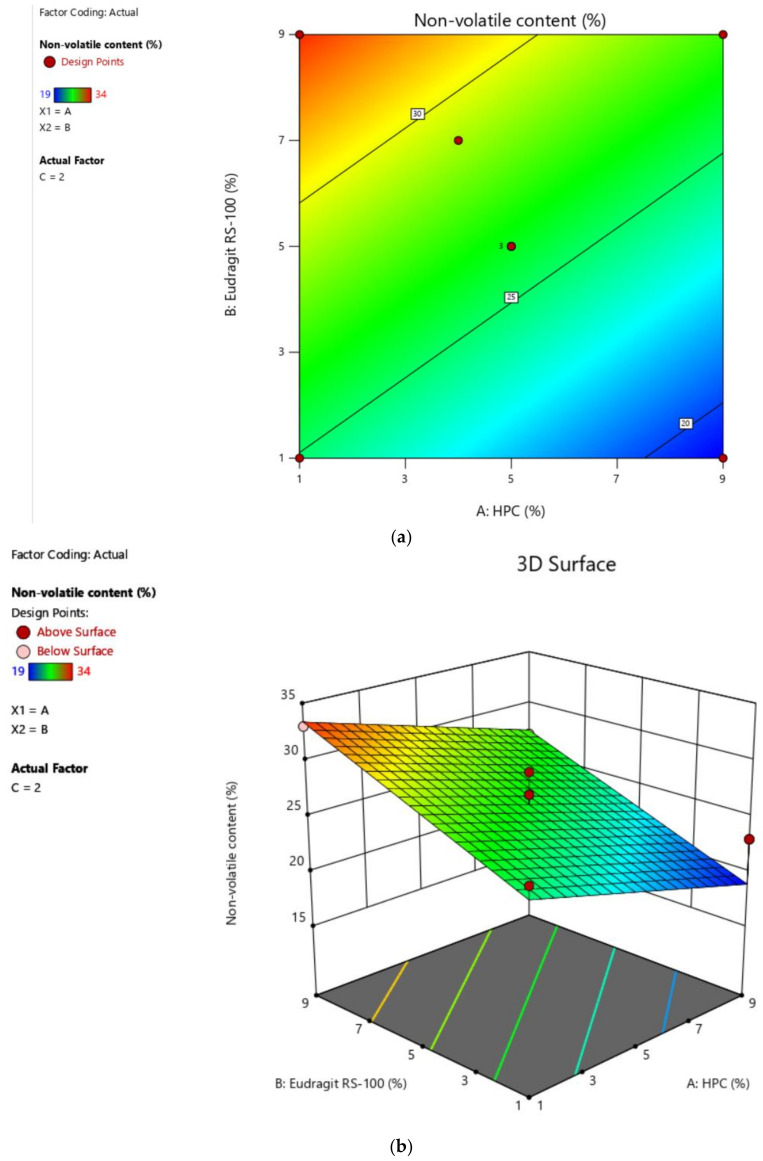
Effect of independent factors on the non-volatile content with contour plot (**a**) and 3-D response surface (**b**) of nail lacquer by the application BBD.

The optimization as conducted through BBD (Table 3 and Figure 4) for percentage non-volatile content, showed the effects of variables which when subjected to ANOVA analyses revealed a quadratic model and F-value of 10.22 (*p* < 0.05) implying that the model is significant.

#### 2.4.3. Water-Resistance Capacity

All formulations responded differently in terms of water resistance capacity. It was shown that the resistivity of water is high for films, which have a high hydrophobic polymer ratio. In contrast, resistivity of water is low for films, which comprised of high hydrophilic polymer ratio. Conclusively, it is noted that the resistivity of water is dependent on the nature of the polymer and the overall hydrophilicity or hydrophobicity of the film composition.

#### 2.4.4. Blush Test for Nail Lacquer

Visual examination of all the films revealed a slight diminution of brightness. The dullness of the films may be attributed to the presence of hydrophilic polymer in all the formulations that could not impart complete resistance to the effect of water on the films. The composition of the films has to be judicious in terms of hydrophobicity and hydrophobicity so as to allow adherence onto the nail surface while still exhibiting adequate water resistance. Almost all formulations exhibited complete blistering or peeling off, which is notably an important formulation attribute.

#### 2.4.5. In Vitro Drug Release Study

**Figure 5:**
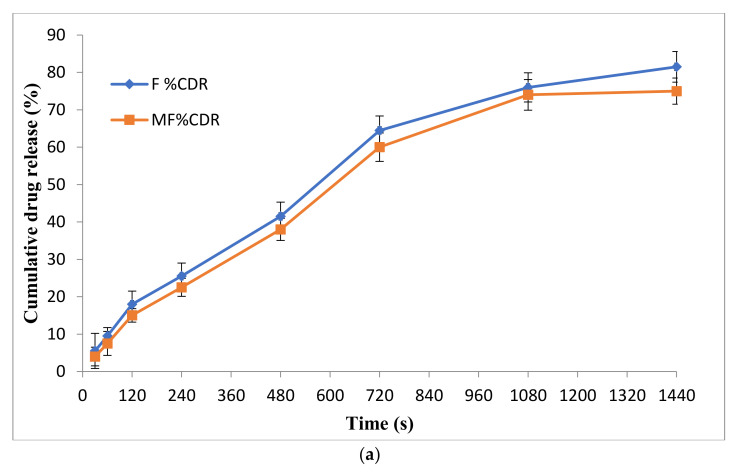

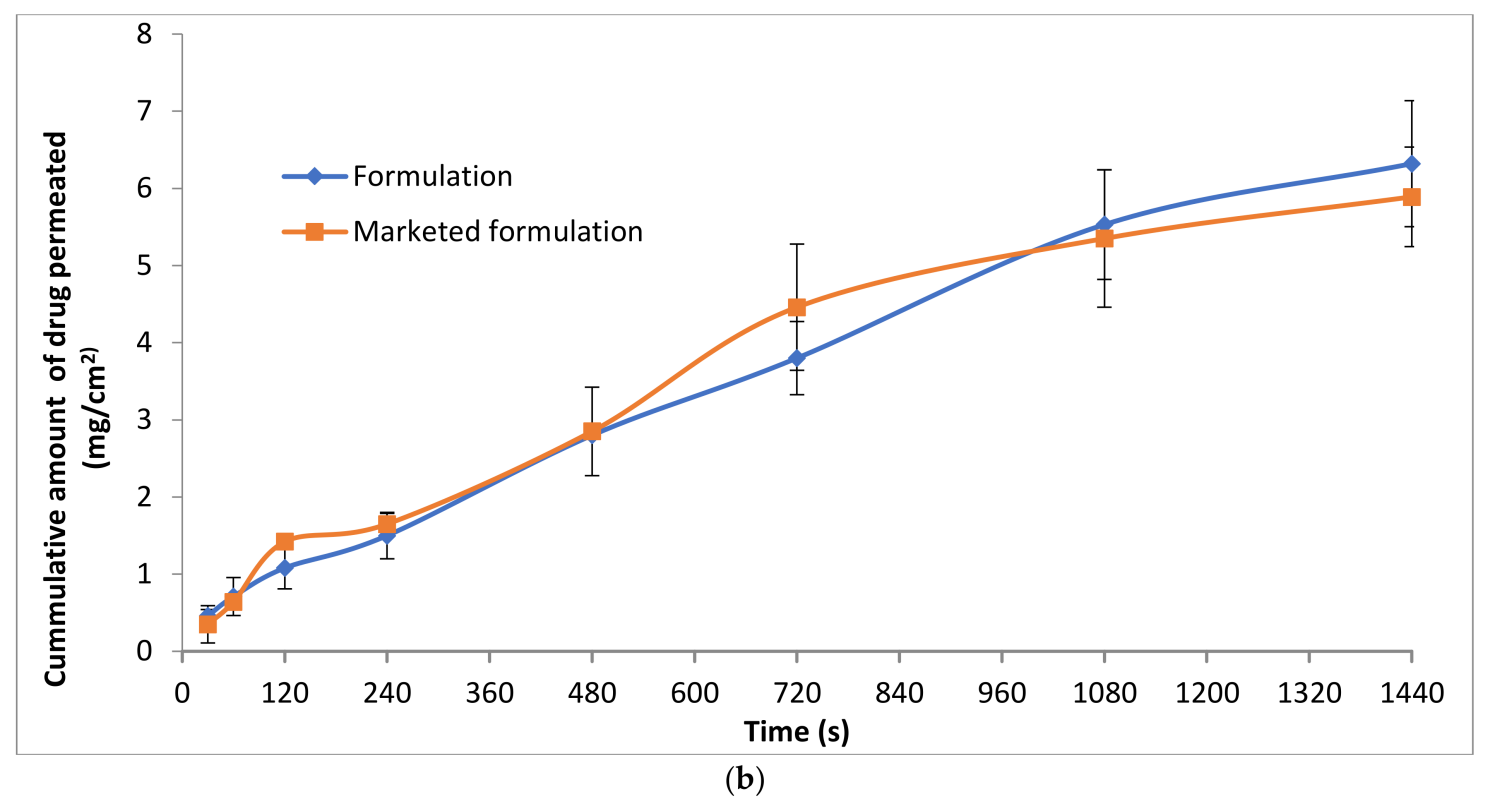
(**a**) Comparison of in vitro release behaviour of optimized formulation with marketed formulation of nail lacquer. (**b**) Transungual permeation studies profile carried on human nail of optimized formulation with marketed formulation of nail lacquer.

An in vitro release study was carried out to check the release of the drug from the formulation compared to marketed preparation (Figure 5a). It was observed that the prepared formulation showed better-sustained release (% CDR 81.5) in comparison with the marketed formulation (% CDR 75). Moreover, the formulation followed a drug release kinetics based on the Higuchi model, with an R2 value of 0.984.

#### 2.4.6. Transungual Permeation Study

A transungual permeation study was carried out to optimize the drug permeation of formulation in comparison with marketed preparation shown in Figure 5b. The cumulative amount of drug permeated from the developed nail lacquer was found to be 6.32 μg/cm^2^ at the end of 1440 s, which was more than the amount of drug permeated from the marketed preparation with a permeation rate of 5.89 μg/cm. However, the drug permeation at the end of 24 h was higher than that of the minimum inhibitory concentration, which was 0.25 μg/mL and 2 μg/mL of the drug, respectively, for the marketed and the optimized formulation Thus, it can be concluded that the prepared nail lacquer formulation could be a promising drug delivery approach for treating onychomycosis and would be successful in combating the fungal infection.

### 2.5. Development of Nanoemulgel

#### 2.5.1. Screening of Excipients

**Figure 6:**
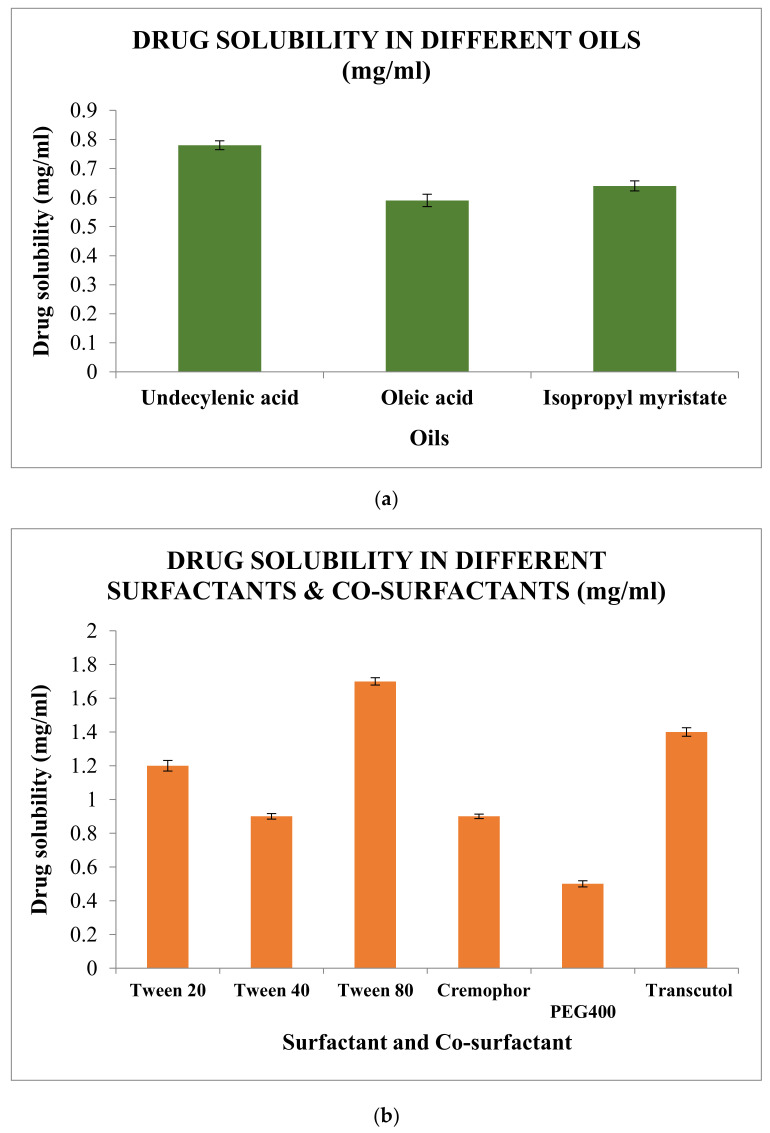
(**a**) Solubility of Amorolfine HCl in different oilsIt was highest in undecylenic acid (0.78 ± 0.0152 mg/mL). (**b**) Solubility in different surfactants (Tween 80-1.7 ± 0.0218 mg/mL) and co- surfactants (Transcutol-1.4 ± 0.0253 mg/mL).

Undecylenic acid (UA) was selected and used both as an oil phase because of the highest solubility of the drug in it (Figure 6a) and also for having a proven antifungal activity [16]. It is a derivative of castor oil, and it is used in various marketed formulations for the treatment of nail and skin infections. Clinical practices have also supported the use of UA in inhibiting the fungal infections with negative fungal culture for prolonged periods of time [17,18,19].

Solubility of Amorolfine HCl in different surfactant and co-surfactant was spectroscopically analysed as mentioned in Section 5.2, and the observations were depicted in the following histogram (Figure 6b). Based on the observations, Tween 80 and Transcutol were selected as surfactant and co-surfactant, respectively.

#### 2.5.2. Development of Nanoemulsion (NE) (o/w type) by Construction of Pseudo-Ternary Phase Diagram

The pseudo-ternary phase diagram was used to set out the optimal composition range for excipients. It is a technique used to engineer NE areas. It shows the impact of volume change of different phases on the behaviour of the system.

**Figure 7:**
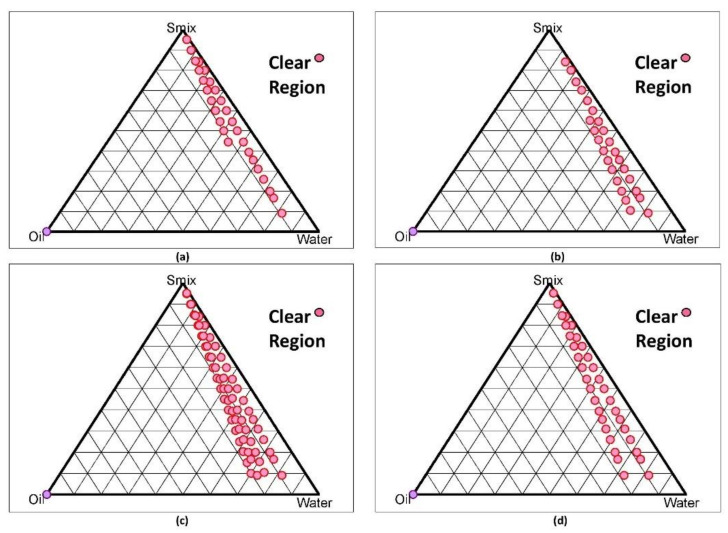
Pseudo-ternary phase diagram of nanoemulsion formulations composed of oil phase (undecylenic acid) and Smix (Tween 80 and transcutol) in ratio of (**a**) 1:1, (**b**) 2:1, (**c**) 3:1, and (**d**) 4:1.

The diagram concluded that out of 1:1, 2:1, 3:1, and 4:1, the pseudo-ternary phase diagram showing oil in water NE region for 3:1 Smix covers the maximum area and hence was selected for further studies (Figure 7).

#### 2.5.3. Thermodynamic Stability Studies

Stability studies of various NEs is summarized in Table 5. Based on the observations in Table 5, selected formulations (F4, F7, F8, F12, and F13) were subjected to further optimization. Their size was analysed by zeta sizer after sonicating NEs for 60 s (2 cycles/60 s). The size for these formulations, F4, F7, F8, F12, and F13, were determined.

Out of all formulations, F7 was selected because of its stability and desired particle size. It was further subjected to different characterization studies.

### 2.6. Characterization of Stable Nanoemulsion

#### 2.6.1. Particle Size and Polydispersity Index (PDI)

**Figure 8:**
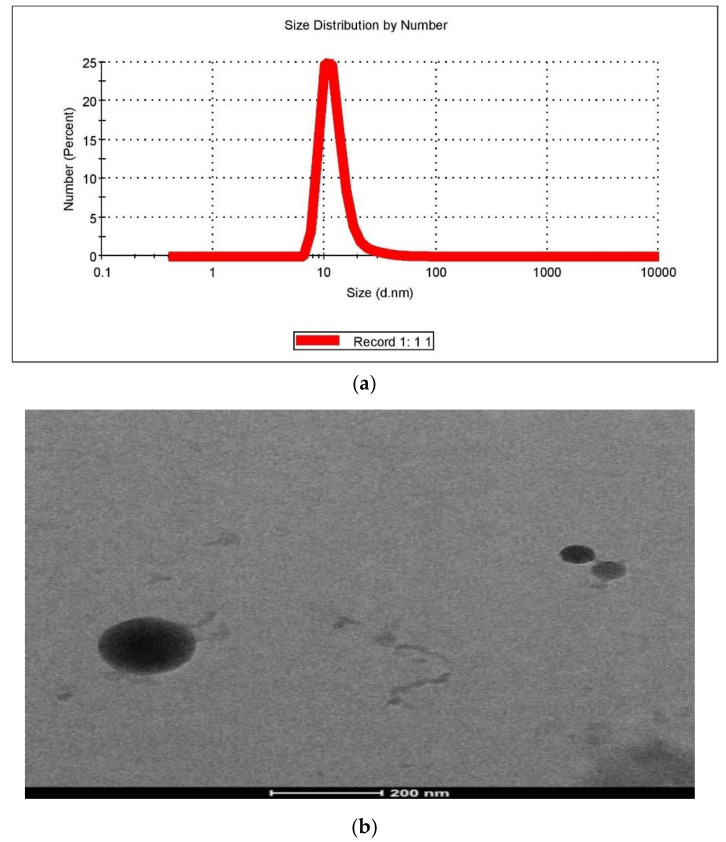
(**a**) Zeta sizing of optimized formulation Amorolfine HCl nanoemulsion (78.04 ± 4.724 nm). (**b**) TEM image of Amorolfine HCl nanoemulsion slightly spherical structure with dimension between 100–120 nm.

NE particle size should be less than 200 nm to exhibit appropriate formulation attributes [20]. The particle size of optimized NE prepared by aqueous titration followed by high-pressure homogenization was found to be in the nanometric range, i.e., 78.04 ± 4.724 nm (Figure 8a).

PDI indicates the size distribution and homogeneity of dispersed particles in a given formulation. PDI values between 0.1 to 0.3 suggest narrow size distribution while a value above 0.4 indicates broad size distribution. A value between 0.10 and 0.40 indicates the system is moderately polydispersed [21].

PDI of optimized NE was found to be 0.274 which indicates that optimized NE is a moderately polydispersed system.

#### 2.6.2. Zeta Potential

Zeta potential analysis is an efficient way to evaluate the stability of a dispersed system. It reflects upon the degree of particle aggregation due to electrostatic repulsion [22]. For the NE preparation, non-ionic surfactants are commonly used as these would expectably maintain stability, compatibility, and toxicity, contrary to ionic or amphoteric surfactants [21].

The zeta potential of the formulation was detected using a DLS technique powered with a computerized inspection system with DTS software and was found to be −10.7mV [23].

#### 2.6.3. Morphology/TEM

TEM analysis of the optimized formulation showed a slightly spherical structure with dimensions between 100–120 nm (Figure 8b). This size was slightly more than what was obtained by Malvern Zetasizer. This difference in particle size might be because of the difference in the principle of analysis and sample preparation as per both methods.

#### 2.6.4. pH

The pH value of the optimized formulation was found to be 6.3 ± 0.24. The assessed pH value would allow the preparation to be skin compatible and would not presumably cause any irritation.

### 2.7. Development of Nanoemulgel

NEGs were formulated using 0.5% to 1.5% *w*/*v* gelling agent, carbopol-934. Table 6 summarizes observations made for NEG with varying percentages of carbopol-934 and thioglycolic acid, which was used as a penetration enhancer (Table 6).

Gel formulated with 0.75% *w*/*v* Carbopol and 1.5% *v*/*v* thioglycolic acid (NEG0.75) was the best and most stable formulation out of all the prepared formulations in terms of appearance, texture, homogeneity, and spreadability. Below this concentration of Carbopol, the gel was quite watery, and above this concentration it became hard.

To the optimized gel formulation, 1% *v*/*v* glycerine was added as a humectant and 0.02% *w*/*v* methylparaben as a preservative. It was further subjected to different evaluation parameters of gel.

### 2.8. Evaluation of Nanoemulgel

#### 2.8.1. Spreadability Study

After spreading the gel, the diameter was found to be 6.8 ± 0.127 cm, indicating the easy spreadability of the gel.

Gel characterizations were done to assess the gel based on various parameters and are summarized in Table 7.

#### 2.8.2. Extrudability Study

The extrudability study determines the amount of gel extruded from the tube on applying a certain weight. A ribbon of at least 0.5 cm should be extruded in 10 s. Further, the gel with better extrudability has more gel extruded from the tube [24].

The extrudability of optimized NEG was found to be 1.7 ± 0.32 gm/cm^2^ which is considered optimal.

#### 2.8.3. Gel Texture Analysis and Stability study

**Figure 9:**
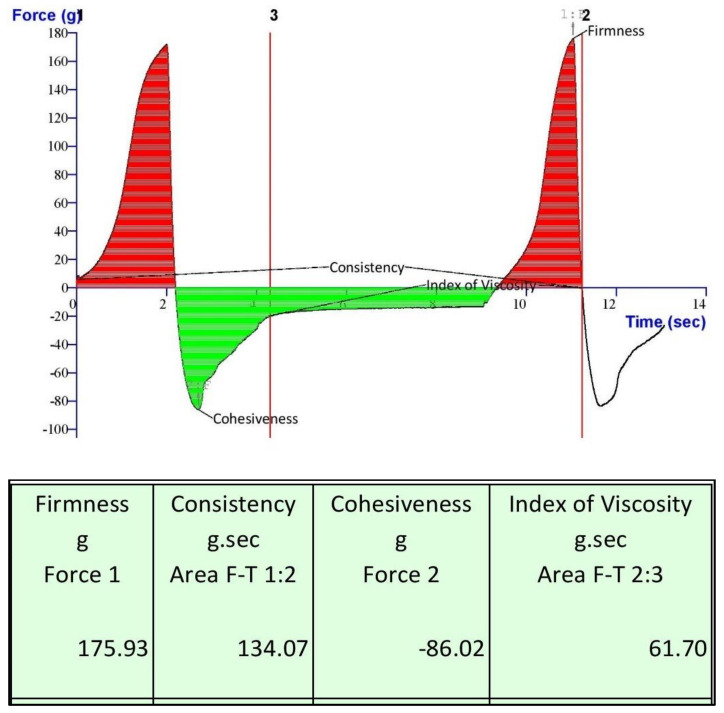
Mechanical properties of Amorolfine HCl nanoemulgel.

Texture analysis of optimized NEG was performed and is shown in Figure 9. Firmness, consistency, cohesiveness, and index of viscosity are some of the mechanical properties of the gel formulation. These properties directly affect the applicability and patient compliance. Viscosity index determines the stickiness of gel, while firmness indicates the strength of the gel. The higher the value, the better the gel strength [25]. The values of firmness, consistency, cohesiveness, and viscosity index of optimized NEG were 175.93 g, 134.07 g/s, −86.02 g, and 61.70 g/s, respectively. The optimized NEG was found to have all the characteristics of the gel.

After 3 weeks, the gel was observed for its stability visually for any signs of breaking and was found to be stable. Other parameters assessed for gel also gave positive results and are recorded in the following Table 7.

#### 2.8.4. In Vitro Drug Release Study

**Figure 10:**
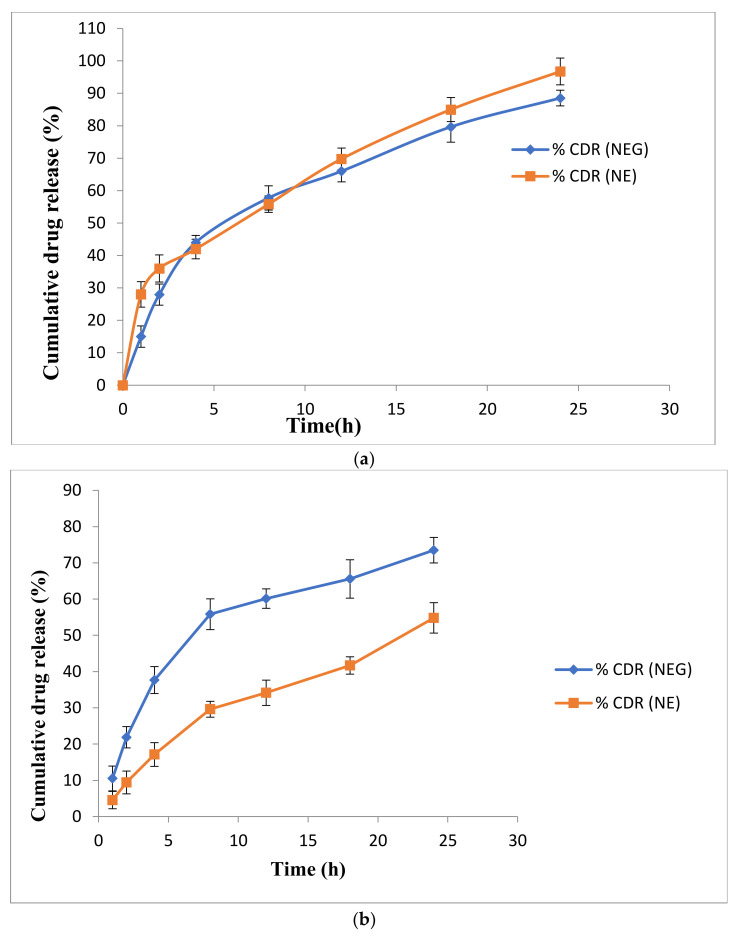
(**a**) Comparison of in vitro release behaviour of Amorolfine HCl NEG and NE following the Higuchi model. (**b**) Transungual permeation studies profile carried on human nail of Amorolfine HCl NEG and NE.

The release of drug from NEG and NE was observed for 24 h, and the in vitro release was plotted between percentage cumulative drug release (percent CDR) vs. time as depicted in Figure 10a. The obtained release data were fitted into various kinetic models to explain the mechanism and kinetics of drug release and find the best fit. The correlation coefficient (R2) value was calculated from the linear curves obtained by regression analysis of the above-said models. The equations were applied for the estimation of the R2 for each model. The model which gave the value of R2 close to 1 was selected [26].

It was observed from the release kinetics data that there was a sustained release pattern from NEG as compared to the NE that showed 36 ± 3.61% release in 2 h, whereas NEG showed 27.94 ± 1.55% release in 2 h. This was because of the three-dimension rigid structure of the NEG. Both followed the Higuchi model as the regression coefficient of this model was close to 1.

#### 2.8.5. Transungual Permeation Study

The release of drug from NEG and NE was observed for 24 h, and an ex vivo graph was plotted between percentage of cumulative drug release (percent CDR) vs. time in Figure 10b. The release data was further evaluated to find steady flux and permeation coefficient for the formulation.

Total drug release from the formulation through human nail was 73.50 ± 0.96 and 54.82 ± 1.97% for NEG and NE, respectively, over 24 h. Cumulative drug release from NEG through cadaver nail was found to be more than NE. This might be due to the presence of a penetration enhancer, thioglycolic acid, that cleaves the disulfide bonds present between the keratin molecule in the nail, thus increasing drug permeation into the nail.

Thioglycolic acid (TA) is a chemical agent that can break down the disulfide bonds present in the keratin of hair, wool, and nails via a redox reaction that affects the rigidity of nail. TA interacts and breaks the disulfide bonds present in the keratin of the nail, and aid in the movement of the drug across the nail plate, thus improving the flux of the formulation [27].

NEG’s ex vivo permeation profile was found to be 374.99 µg/cm^2^, which is higher than the permeation profile of NE, i.e., 279.69 µg/cm^2^. This is the quantity of drug permeated through the nail in 24 h.

A flux of 174.67 µg/cm^2^/h and 93.297 µg/cm^2^/h was calculated for NEG and NE, respectively. The permeation coefficient was calculated using the formula Flux/Total amount of drug in donor apartment (i.e., 500 µg Amorolfine HCl). It was found to be 0.3493 and 0.1866 for NEG and NE, respectively.

The flux and permeation coefficient of NEG were found to be higher than the flux and permeation coefficient of NE, indicating high solubility and high permeation of NEG formulation into the nail, which is a primary requirement for treating onychomycosis. It was also observed that both NE and NEG supported a drug release profile, which ensured that the amount of drug remained well above the minimum inhibitory concentration against the fungi and would help ameliorate onychomycosis.

## 3. Conclusions

Molecular docking has recently emerged as a dependable tool for the selection of drug candidates that could specifically interact with the target protein at the site of action. Based on this, Amorolfine HCl surfaced as a drug of choice for the treatment of onychomycosis. The disease manifests itself in myriad ways and progresses from mild to severe category. Each stage of the disease requires a specifically customized antifungal-laden and locally applied drug delivery system that could ensure the drug’s sustained release well above the MIC and support effective disease amelioration. In this context, the current research attempted to develop an assortment of drug delivery systems with optimal pharmacotechnical attributes like ease of application, spreadability transungual permeation, in vitro drug release profiles, etc. The optimized formulations were fortified with OTC ingredients like vitamin E and undecylenic acid, which support overall nail health. The formulations consisted of nail lacquer with vitamin E and NEG with undecylenic acid, which were found to be amenable to application on the diseased nail surface with prolonged periods of drug release. The advantage of the availability of different types of optimized formulation gives freedom to the treating physician. It helps in prescribing the most appropriate delivery system as per the patient’s need. In conclusion, a customized formulation design is expected to improve patient compliance and, eventually, better disease management.

## 4. Materials

Amorolfine HCl was obtained as a gift sample from Sun-pharma pharmaceuticals Ltd. Gurgaon, India. Eudragit^®^ RS 100 and Hydroxypropyl cellulose were obtained from Nano-tech Research Laboratory Pvt. Ltd. (New Delhi, India). Undecylenic acid, vitamin E, and Cremophor were purchased from Sigma-Aldrich (Darmstadt, Germany). Carbopol 940, Tween 20, Tween 40, Tween 80, and PEG400 were procured from SD fine chemicals (Mumbai, India). Transcutol was procured from Gattefosse (Lyon, France). All excipients and chemicals used were of analytical grade and used without any further purification.

## 5. Method

### 5.1. Molecular Docking Study

This study established diverse interactions between the test compounds and the target protein [28,29]. Here, the molecular docking study was conducted on the 3D structure of the protein (Keratin) with the help of the Maestro 10.5 program (Schrodinger Inc. New York, NY, USA) by using the 64-bit operating system on windows 7 with an HP computer (Intel (R) Core (TM) i5-2400 CPU-2.40 GHz, 6 GB RAM). For this study, the X-ray crystal structure of human keratin (1-Keratin 10 Helix 2B Heterodimer) was obtained from the Protein Data Bank (PDB ID: 4ZRY). In the first step, the obtained protein was prepared with the help of the protein preparation wizard module, and then water and all other objectionable residues were removed except the unique ligand. After this, it was exposed to the optimization of hydrogen bond followed by energy minimization. Then, the active site was generated as a grid box via Glide. The Mol file was prepared using the software Chem Draw 12.0 to draw the structure of ligand molecules, and the energy minimization was done using the LigPrep module of Maestro. After that, at pH 7.0 ± 2.0, all probable ionization states were created and minimized. Then, by using Glide, all the prepared ligand molecules were docked into the active site in extra precision mode (XP). At this point, a G-score value was used to determine the most appropriate compound for targeting, which was responsible for the experimental activity and interaction of the compound (hydrogen bonds, pi–pi interactions, and hydrophobic interactions) [30,31].

### 5.2. Analytical Method Development

The UV–Visible spectrophotometric method was developed using a UV–Visible spectrophotometer (UV-1601/Shimadzu Corporation, Kyoto, Japan) and validated in methanol to estimate drug content and in Phosphate Buffer Saline (PBS) pH 7.4 for drug release studies from the developed formulation. The wavelength (ʎ max) in methanol was 213 nm, and for PBS pH 7.4, it was 220 nm [32].

### 5.3. Development of Nail Lacquer

#### 5.3.1. Selection of Solvent System Ratio and Optimization Polymer Concentration by Application of BBD to Check the Drying Time and Non-Volatile Content

Characteristics of Hydroxypropyl cellulose (HPC) and Eudragit RS are different. HPC is hydrophilic where Eudragit is hydrophobic in nature. HPC is soluble in water but in higher amount HPC starts forming lumps in water which can cause the formulation. To avoid this lump and make Eudragit also soluble, ethanol: water is taken as a ratio of solvent and optimized. This solvent ratio is compatible for both of the polymers.

Drug-free formulations were prepared in which the ratio of both the polymers HPC and Eudragit^®^ RS 100 were varied as 1:1, 1:2, and 2:1, and the ratio of solvent system, in other words, ethanol and water was varied from 9:1, 8:2, and 7:3. An area of 5 cm × 5 cm was marked on a glass Petri dish, and a uniform layer (100 µL) of nail lacquer was spread with the help of a nail lacquer brush. The drying time of the formulation was optimized [33].

The two different categories of polymers were evaluated for the evaporation time and water-resistance capacity by using Hydroxypropyl cellulose (hydrophilic Polymer) and Eudragit^®^ RS 100 (hydrophobic Polymer). The polymers’ different ratios (9:1, 8:2, 7:3, 6:4, 5:5, ……, 1:9) were used to evaluate the evaporation time and water resistivity to further optimize the concentration of polymers for the final formulation [34].

Design Expert 13.0.3.0, an experimental design with response surface morphology of BBD, was used to optimize the polymers and permeation enhancer [35]. Considered independent variables were HPC concentration (percent *w*/*v*), Eudragit^®^ RS 100 (percent *w*/*v*), and Permeation Enhancer (percent *w*/*v*), and dependent variables were non-volatile content (percent *w*/*w*) and drying time (seconds) [36].

#### 5.3.2. Preparation of Nail Lacquer

Accurately weighed amount of hydrophobic polymer was dissolved in a one-half quantity of ethanol (90% *v*/*v*) using an ultrasonicator. In the other half quantity of solvent, the required quantity of drug (5% *w*/*v*), Vit E (0.5% *w*/*v*), and plasticizer (2% *w*/*v*) were dissolved. Then, with the help of a magnetic stirrer, the drug solution was admixed with the polymeric solution. Lastly, a permeation enhancer was added to the prepared mixture of drug and polymer with continuous stirring. Then, the final formulation was stored in an airtight amber-coloured glass container for further use [33].

### 5.4. Evaluation of Nail Lacquer

#### 5.4.1. Drying Time

The volatile characteristics of the solvent system and its drying rate are the significant determinants for nail coating. Here, a thin layer of nail lacquer was allowed to spread on a clean and dry Petri plate (glass) and was observed for a few minutes. Then, with the help of a stopwatch, the time to form a dry-to-touch film was noted down. A dry-to-touch state is a state where there is no deposit of materials on the fingers after touching the film with a clean fingertip [37].

#### 5.4.2. Non-Volatile Content

A 1 ± 0.2 g portion of the sample was weighed and uniformly spread on a Petri-dish, and then placed in a hot air oven at 105 ± 2 °C for 1 h, allowed to dry, and then re-weighed. The difference in weight was determined to calculate the non-volatile content (Bureau of Indian Standard, IS 9445: 1994) [38].

The difference in weight was calculated using the following formula:

% Non-volatile content = (wet weight − dry weight)/(dry weight) × 100

#### 5.4.3. Water-Resistance Capacity

Here, 1 ± 0.2 g of sample was taken and allowed to spread evenly on a glass plate and dried at a temperature of 25 ± 2 °C Then, the weight of the glass plate was taken and immersed in a water bath (HICON, New Delhi, India) previously maintained at a temperature of 37° ± 2 °C. After 24 h, the plate was removed, wiped using tissue paper, and re-weighed. The variation of weight was reported in terms of high or low [39].

#### 5.4.4. Blush Test for Nail Lacquer

A 0.2 g sample was poured over a tin plate and spread evenly. It was then kept for drying at room temperature for 24 h. A glass beaker (250 mL) was half-filled with ordinary tap water, and the plates were dipped in the beaker in such a way that half of the coating is in water and the remaining portion is above water which allowed to remain as such for 4 h. The plates were then removed, wiped with tissue paper, dried at room temperature for 4 h, and checked for the presence of any blush. The material was said to pass the test if it had no or slight whitishness. The film should not show any blistering or peeling off [40].

#### 5.4.5. In Vitro Release Study

In vitro drug release study was carried using dialysis membrane-60 (average diameter—16mm, average width 25 mm, Himedia^®^, Mumbai, India). Firstly, the humectant glycerol was removed by washing the membrane under running water for 3–4 h. Then, it was treated with 0.3% *w*/*v* sodium sulfide solution at 80 °C for 1 m to get rid of sulphur compounds. It was after that washed with hot water at 60 °C for 2 min, followed by acidification with a solution of 0.2% (*v*/*v*) H_2_SO_4_. It was further rinsed with hot water to remove the acid [41]. Meanwhile, 500 mL phosphate buffer pH 7.4 was prepared as per IP 2018.

Franz diffusion cell was used to carry out the in vitro release study of the optimized formulation [42,43]. About 100 microlitres of formulation sample (drug-loaded dual-component nail lacquer) were applied over a dialysis membrane that was fitted over the Franz diffusion cell. The membrane was fixed in a way that it only touched the surface of the release media filled in the doner cell placed over a magnetic stirrer maintained at a temperature of 37 ± 2 °C, and a stirring speed of 100 rpm. One millilitre aliquots of the dissolution media were withdrawn at a fixed time interval which was replaced with a new dissolution media.

Then, the sample solution containing Amorolfine HCl was filtered through a 0.22 μm membrane filter, analysed spectroscopically at 220 nm by the method mentioned in Section 5.2. The same procedure was repeated for the marketed formulation. Lastly, elucidation of the release mechanism was done by fitting the in vitro release data to zero order, first order, Higuchi model, and Korsmeyer–Peppas model [32].

#### 5.4.6. Transungual Permeation Study

Human nails were collected from the local salon and allowed to soak in distilled water for 24 h. A section of about 1mm thickness was cut from the lower part of the nail using a microtome, which was then kept between the donor and receptor compartment of Franz diffusion cell with an effective surface area of 1.23 cm^2^ and receptor cell volume 10 mL. A total of 100 microliters of the formulation sample (drug-loaded a dual component nails lacquer) was applied carefully in the donor compartment of the Franz diffusion cell. The receptor compartment was filled with the solvent system (PBS pH 7.4), and the whole assembly was maintained at 37 ± 2 °C with constant stirring at 100 rpm for 24 h. Samples were withdrawn (2 mL) at an appropriate interval (0, 30 min, 1, 2, 4, 8 h.) through a sampling port, filtered, and analysed spectroscopically by the method mentioned in Section 5.2. Sink conditions were maintained throughout the experiment. The cumulative amount of drug permeated per unit area (CDP/A) through the human nail plate was plotted against time [37,44,45] to obtain the in vitro release profile.

### 5.5. Development of Nanoemulgel

#### 5.5.1. Screening of Excipients

The selection of oil, surfactants, and co-surfactants was done based on the drug’s solubility. An amount of 1 mL of each of the oils, surfactants, and co-surfactants were taken into 5 mL glass vials, and to this excess amount of drug was added. The samples were vortexed and maintained at 37 ± 2 °C for 72 h in an incubator shaker at 100 RPM. Then, the upper layer of the solution was taken, and suitable dilution was made using methanol which was further analysed using a developed analytical method. Oil, surfactants, and co-surfactants showing the highest amount of dissolved drug were selected [46].

#### 5.5.2. Development of Nanoemulsion (NE) (Oil in Water Type) by Constructing Pseudo-Ternary Phase Diagram

With the help of the aqueous titration method, a pseudo-ternary phase diagram was constructed by using Smix of Tween 80: transcutol in different proportions such as 1:1, 2:1, 3:1, and 4:1. Oil phase (Undecylenic acid) and S mix were then mixed at different weight ratios of 1:9, 2:8 (1:4), 3:7 (1:2.3), 4:6 (1:1.5), 5:5 (1:1), 6:4 (1:0.7), 7:3 (1:0.43), 8:2 (1:0.25), 9:1 (1:0.1), 1:2, 1:3, 1:3.5, 1:5, 1:6, 1:7, and 1:8 (*w*/*w*), to cover the maximum proportions for the study. These weight ratios of oil and S mix were then diluted drop-wise under moderate agitation. After equilibration, the mixtures were visually evaluated and determined as NEs by their clarity, transparency, and flowability [47].

#### 5.5.3. Thermodynamic Stability Studies

A screening method was applied to find out the best and most stable formulation. The NEs were subjected to the following stress–stability studies.

Heating–Cooling Cycle: Selected formulations were exposed to three heating and cooling cycles over a temperature range of 4–45 °C; they were stored for 48 h at each temperature and then were observed for any physical instability such as flocculation, cracking phase separation, and precipitation.

Centrifugation: Formulations that passed the previous test were then subjected to centrifugation at 3500 rpm for 30 ms to discard metastable system and were observed for any changes in homogeneity.

Freeze–Thaw Cycle: Lastly, the formulations were kept between the temperature range of −21 °C and + 25 °C for 48 h, up to 3 cycles. Any changes in the homogeneity of emulsions were observed [48].

### 5.6. Characterization of Stable Nanoemulsion

#### 5.6.1. Particle Size and Polydispersity Index (PDI)

Size and PDI were determined using a zeta sizer that is based on the principle of dynamic light scattering (DLS) technique using a computerized inspection system (Malvern Zetasizer, Nono-ZS, Malvern) carrying DTS software. For particle size, the formulation was diluted with distilled water which was then placed in a quartz cuvette and subjected to the size analysis (n = 3) [49].

#### 5.6.2. Zeta Potential

Zeta Potential was measured using a zeta sizer (n = 3) (Malvern Zetasizer, Nono-ZS, Malvern, Malvern, UK) that has the same principle as that of particle size determination. It is used for surface charge analysis [50].

#### 5.6.3. Morphology/TEM

Transmission electron microscopy (TEM) (TECNAI G2 200kV, HR-TEM from FEI Company, Eindhoven, The Netherlands) was used for surface characteristics of the NE. A droplet of the NE, which was previously diluted, was applied on a farmvor-coated copper grid and treated with a 2% aqueous solution of phosphotungstic acid (PTA) and dried for 30 s, and then the grid was taken on a slide and covered with a cover-slip, before examining under a microscope [51].

#### 5.6.4. pH

The pH value of NE was determined using a calibrated pH meter (Mettler Toledo MP 2020, Mumbai, India) at 25 °C (n = 3).

### 5.7. Development of Nanoemulgel (NEG)

For formulating NEG, gel base was prepared by dispersing gelling agent (0.5% to 1.5% *w*/*v*) in NE and constantly stirring using a magnetic stirrer at a moderate speed, penetration enhancer (1% to 2% *v*/*v*) was also added [52].

Accurately weighed amount of gelling agent (Carbopol-934), which was previously passed through sieve no. 40, was added in little quantity with continuous stirring. The temperature of the stirrer was maintained at 60 ± 2 °C until the whole of the Carbopol was incorporated. To this, the desired quantity of thioglycolic acid (penetration enhancer) was added. The mixture was left overnight and then neutralized by adding triethanolamine (0.056% *v/v*) drop-wise until gelling was achieved. The prepared NEG formulations were then examined for colour, appearance, viscosity, and consistency [24].

### 5.8. Evaluation of Nanoemulgel

#### 5.8.1. Spreadability Study

Spreadability was determined by 1 g gel placed on a round glass slide, fixed on a wooden block, and sandwiched using a similar slide. The increase in the diameter of the gel was noted after five minutes of placing another glass slide of 500 g [53].

#### 5.8.2. Extrudability Study

Extrudability Study is a test to measure the force required to extrude the gel from the tube [54]. The extrudability of optimized formulation was calculated by using the formula:

E = M/A

where E = Extrudability; M = Applied weight to extrude gel from the tube; A = Area.

#### 5.8.3. Gel Texture Analysis and Stability Study

The gel was stored for 3 weeks at room temperature and was visually observed for any sign of breaking, texture, and other rheological parameters [53]. Different rheological studies of gel were performed to assess parameters like texture, homogeneity, pH, and drug content. Texture analysis of optimized NEG was performed using Texture analyser TA-XT Plus (Stable Micro Systems, Godalming, UK). Drug content was analysed by taking 1 mg gel, extracting the gel in methanol, and was analysed using the method mentioned in Section 5.2.

#### 5.8.4. In Vitro Drug Release Study

In vitro drug release study was performed as per the procedure mentioned in Section 5.4.5. About 1 g of NEG was applied over a dialysis membrane which was fitted over the Franz diffusion cell. The whole system was maintained at 37 ± 2 °C and 100 rpm for 24 h. A 1 mL drug sample was withdrawn at pre-determined time intervals (0, 1, 2, 4, 8, 12, 18, 24 h), filtered through a 0.22 μm membrane filter, and replaced with an equal quantity of fresh solvent to maintain the sink conditions throughout the experiment. The sample taken out was analysed spectroscopically at 220 nm, as mentioned in Section 5.2. for the amount of drugs present. The same procedure was repeated for the NE formulation [54,55].

Release kinetic modelling was done by putting the in vitro release data into zero-order, first-order, Higuchi and Korsmeyer–Peppas release kinetics model. The model that represented the highest value of correlation coefficient was considered as the best fit model.

#### 5.8.5. Transungual Permeation Study

Transungual permeation study was performed as per the procedure mentioned in Section 5.4.6. About 1 g NEG was placed between the donor and receptor compartments, receptor compartment was filled with PBS pH 7.4 (9 mL). The whole assembly was maintained at 37 ± 2 °C, and the stirring speed was kept constant at 100 rpm for 24 h. An amount of 1 mL of the sample was withdrawn at pre-determined time intervals (0, 1, 2, 4, 8, 12, 18, and 24 h), filtered through a 0.22-μm membrane filter, and replaced with an equal quantity of fresh solvent. The sample taken out was analysed for the amount of drug present using the spectroscopical method mentioned in Section 5.2. The same procedure was repeated for the NE formulation [56].

## Figures and Tables

**Table 1:** International status of some commercial products available in markets such as the USA and EU.

S. No.	Trade Name	Composition	Manufacturing Authorization Holder & Product Category	Indication	NDA No/Weblink
1	Loceryl (Nail Lacquer)	5% *w*/*v* Amorolfine in HCl	Galderma (UK), PoM	Onychomycoses caused by dermatophytes, yeasts and moulds.	https://www.medicines.org.uk/emc/product/1411 (accessed on 11 July 2021)
2	Penlac (Nail Lacquer)	Ciclopirox 8%	Valeant Bermuda (USA), PoM	Immunocompetent patients with mild to moderate onychomycosis due to Trichophyton rubrum	NDA-021022
3	Kerydin (Nail solution)	Tavaborole 5%	Anacor Pharmaceuticals Inc., (USA), PoM	Onychomycosis of the toenails due to Trichophyton rubrum or Trichophyton mentagrophytes	NDA-204427
4	Trosyl (Nail solution)	Tioconazole 283 mg/mL	Pfizer (UK), PoM	Nail infections due to susceptible fungi (dermatophytes and yeasts) and bacteria.	https://www.medicines.org.uk/emc/product/1071/smpc (accessed 11 July 2021)
5	JUBLIA (Nail solution)	Efinaconazole 10%	DOW PHARM (USA), PoM	Onychomycosis of the toenails due to Trichophyton rubrum and Trichophyton mentagrophytes	NDA 203567
6	Danipro (Nail polish)	Undecylenic Acid, B7, Vit E, Vit A	Danipro (OTC)	Several benefits owing to the presence of different components	https://danipronails.com/ (accessed on 11 July 2021)
7	Myco Nail (Nail Lacquer)	Undecylenic acid 25%	Kramer Novis (OTC)	Athlete’s foot (Tinea pedis), ringworm (tinea corporis). For effective relief of itching, irritation, and burning feet.	www.drugs.com/otc/129363/myco-nail-a-antifungal-solution.html (accessed on 11 July 2021)

**Table 2:** Lowest binding energy for the ligand–keratins (PDB; 4ZRY) interaction, along with scores for various interaction types, as detected by GLIDE.

Compounds name	G-Score	Lipophilic E vdw	H-bond	Electro	Protein Ligands Interaction
Amorolfine HCl	−7.325	−5.29	−0.45	−0.14	Tyr A:449, Leu A:452, Leu A:453, Glu A:456, Tyr B:482, Leu B:485, Leu B:486, Glu B:487, Gly B:488, Glu B:489
Ciclopirox	−6.668	−4.65	−0.65	0.22	Tyr A:449, Leu A:452, Leu A:453, Gly A:455, Leu B:485, Leu B:486
Efinaconazole	−5.033	−5.29	−0.53	−0.11	Tyr A:449, Leu A:452, Leu A:453, Glu A:456
Tioconazole	−4.200	−5.30	0	0.03	Tyr A:449, Leu A:452, Gly A:455, Glu A:456
Tavaborole	v3.769	−4.74	0	0.02	Tyr A:449, Leu A:452, Leu B:485

**Table 3:** BBD application to optimize independent variables responses of different nail lacquer formulation’s batches by 32 factorial designs.

	Factors			Response	
Drug Free Formulation (DFF)	HPC (% *w/v*)	Eudragit RS (% *w/v*)	Salicylic Acid (% *w/v*)	Drying Time (s) ± SD (n = 3)	Non-Volatile Content (%) ± SD (n = 3)
DFF 1	5	5	2	220 ± 0.89	27 ± 0.09
DFF 2	1	1	2	160 ± 0.65	26 ± 0.08
DFF 3	5	9	5	340 ± 0.32	34 ± 0.18
DFF 4	5	5	2	220 ± 0.58	27 ± 0.12
DFF 5	9	1	2	240 ± 0.84	23 ± 0.11
DFF 6	9	5	5	280 ± 0.89	24 ± 0.05
DFF 7	5	1	5	260 ± 0.64	22 ± 0.06
DFF 8	1	5	1	220 ± 0.69	28 ± 0.13
DFF 9	1	5	5	280 ± 0.75	30 ± 0.09
DFF 10	1	9	2	340 ± 0.95	33 ± 0.11
DFF 11	4	7	2	200 ± 0.83	29 ± 0.09
DFF 12	9	5	1	190 ± 0.51	19 ± 0.11
DFF 13	9	9	2	280 ± 0.63	27 ± 0.05
DFF 14	5	5	2	200 ± 0.93	29 ± 0.06
DFF 15	5	5	3	180 ± 0.72	21 ± 0.08
DFF 16	5	4	1	160 ± 0.37	23 ± 0.10
DFF 17	5	9	1	300 ± 0.96	32 ± 0.04

After optimizing the polymer and permeation enhancer concentration as independent factors for formulation through BBD, six formulations were chosen from 17 formulations run, based on different concentration to optimize the best formulation. The following Table 4 depicts the quality of the film of six selected nail lacquer formulations after their development.

**Table 4:** Quality of film observes in different developed formulations for drug-free a dual component loaded nail lacquer.

Formulation Code (F)	HPC Conc. (% *w*/*v*)	Eudragit RS 100 (% *w*/*v*)	Ethanol:Water (9:1) mL	Quality of Film
F1	5	5	5	Excellent pliable
F2	9	5	5	Poor inflexible
F4	1	5	5	Good rigid
F6	9	9	5	Very bad
F9	9	1	5	Very easy to tear
F11	1	9	5	Hard

**Table 5:** Stability studies observations of different Amorolfine HCl nanoemulsion formulations.

Smix	Formulation No. (F)	Turbidity	After 24 h Turbidity	Heating-Cooling Cycle	Centrifugation	Freeze Thaw Cycle
1:1	F1(1:9)	No	No	Fail	-	-
2:1	F2(1:9)	No	No	Fail	-	-
2:1	F3(1:8)	Yes	Yes	Pass	Pass	Fail
3:1	F4(1:9)	No	No	Pass	Pass	Pass
3:1	F5(2:8)	No	Yes	-	-	-
3:1	F6(3:7)	No	Yes	-	-	-
3:1	F7(1:5)	No	No	Pass	Pass	Pass
3:1	F8(1:6)	No	No	Pass	Pass	Pass
3:1	F9(1:7)	No	No	Pass	Pass	Fail
3:1	F10(1:3.5)	No	Yes	-	-	-
4:1	F11(1:9)	No	No	Fail	-	-
4:1	F12(1:8)	No	No	Pass	Pass	Pass
4:1	F13(1:7)	Yes	Yes	Pass	Pass	Pass

**Table 6:** Observations of different Amorolfine HCl nanoemulgel formulations.

Formulation (Nanoemulgel = NEG)	Carbopol (% *w*/*v*)	Thioglycolic Acid % (*v*/*v*)	pH	Homogeneity	Spread Ability
NEG 0.5	0.5	1	6.3 ± 0.29	Good	Watery
NEG 0.75	0.75	1.5	6.4 ± 0.21	Better	Good
NEG 1.0	1	1.75	6.4 ± 0.18	Coarse	Hard
NEG 1.5	1.5	2	6.4 ± 0.32	Hard	Hard

**Table 7:** Gel character analyses of Amorolfine HCl nanoemulgel.

S. No.	Parameters (Mean ± SD) (n = 3)	Values
1.	Spreadability (cm)	6.8 ± 0.127 (Easily spreadable)
2.	Homogeneity	Smooth texture (no grittiness was found)
3.	Extrudability (gm/cm^2^)	1.7 ± 0.32
4.	pH	6.4 ± 0.324
5.	Drug Content (%)	94.65 ± 0.43
